# Similar fixation of total knee arthroplasty components with medial congruent and cruciate retaining polyethylene inserts. A randomised double‐blinded controlled radiostereometry trial with 24 months of follow‐up

**DOI:** 10.1002/ksa.12753

**Published:** 2025-07-07

**Authors:** Carl Christian Holkgaard Burvil, Karina Nørgaard Linde, Maiken Stilling, Torben Bæk Hansen, Jesper Dalsgaard, Søren Rytter, Daan Koppens, Emil Toft Petersen

**Affiliations:** ^1^ AutoRSA Research Group, Orthopaedic Research Unit, Aarhus University Hospital Aarhus N Denmark; ^2^ Department of Clinical Medicine Aarhus University Aarhus Denmark; ^3^ Department of Orthopedic Surgery Aarhus University Hospital Aarhus N Denmark; ^4^ University Clinic for Hand, Hip and Knee Surgery, Gødstrup Hospital Gødstrup Denmark

**Keywords:** medial congruent, migration, radiostereometry, RSA, total knee arthroplasty

## Abstract

**Purpose:**

To assess the influence of a higher constraint medial congruent (MC) versus a cruciate retaining (CR) (gold standard) polyethylene insert on the migration of cemented tibial and femoral components of the Persona Total Knee System. High‐constraint inserts may increase forces on components and potentially affect implant migration and long‐term fixation.

**Methods:**

A cohort of 66 patients with primary knee osteoarthritis received a cemented Persona total knee arthroplasty and were randomised to either an MC or a CR polyethylene insert. Tibial and femoral component migration was assessed using static radiostereometric analysis, with the first stereo‐radiograph taken supine on the first postoperative day (baseline) and again at 3‐, 12‐, and 24‐month follow‐up. Tibial and femoral component migration was evaluated in six degrees of freedom and as maximum total point motion (MTPM).

**Results:**

At the 12‐month follow‐up, the mean difference in tibial component MTPM was 0.04 mm (95% confidence interval [CI]: −0.21 to 0.28). The mean MTPM was 0.81 mm (95% CI: 0.64–0.99) in the MC group and 0.85 mm (95% CI: 0.68–1.03) in the CR group. Signed migrations were similar for the MC and the CR group throughout 24 months of follow‐up (*p* > 0.09). At the 12‐month follow‐up, the mean difference in femoral component MTPM was 0.18 mm (95% CI: −0.17 to 0.53). The mean MTPM was 1.08 mm (95% CI: 0.83–1.32) in the MC group and 1.26 mm (95% CI: 1.01–1.51) in the CR group. The femoral components with MC insert had 0.18 mm (95% CI: 0.03–0.32) and 0.20 mm (95% CI: 0.01–0.39) more lateral migration at 3‐ and 12‐month follow‐up, respectively. At the 24‐month follow‐up, there was no statistically significant differences in migration for either translations, rotations or MTPM.

**Conclusions:**

Both tibial and femoral components had similar and acceptable fixation regardless of the type of polyethylene insert.

**Level of Evidence:**

Level I.

**Abbreviations**
CAD: computer aided design
CN: condition number
CR: cruciate retaining
FJS: forgotten joint score
KOOS: knee injury and osteoarthritis outcome score
MC: medial congruent
ME: mean error
MIC: minimal important change
MTPM: maximal total point motion
OKS: oxford knee score
PROMs: patient reported outcome measurements
ROM: range of motion
RSA: radiosterometry
SD: standard deviation
TKA: total knee arthroplasty

## INTRODUCTION

New prosthetic designs in total knee arthroplasty (TKA) that mimic the natural knee biomechanics may improve knee stability, function, and patient satisfaction [41]. The Persona Total Knee System (Zimmer Biomet, Warsaw, Indiana, USA) provides an asymmetric medial congruent (MC) polyethylene insert and a classic symmetric cruciate retaining (CR) polyethylene insert. The MC insert has a conformity of the medial joint compartment, which is higher with a taller anterior polyethylene lip and a more posterior femoral component dwell point [31]. The increased conformity in the MC insert ensures better component congruency during gait, which imitates the kinematics of a native knee better than the CR insert [31]. The native kinematics of the MC knee can potentially be advantageous in more demanding recreational activities. This is particularly relevant considering the increase in TKA's performed in younger patients [5, 21, 39]. In support hereof, indications of higher patient satisfaction with an MC over a CR polyethylene insert have been reported [9].

The higher conformity of the MC insert may enhance constraints within the knee arthroplasty, potentially leading to higher forces on the implant components, which could influence fixation. Early migration of the tibial components in TKA can be measured precisely with radiostereometric analysis (RSA) and has been shown to have a high predictive value (up to 85%) for aseptic loosening and late revision surgery [11, 34, 37]. In younger patients, who have longer life expectancy, ensuring long‐term fixation of the implant is critical. Two RSA studies have investigated MC versus CR influence on implant migration for the Persona Knee and found no difference [7, 26]. However, more evidence is needed to deem the safety regarding fixation with MC components.

The primary aim of this study was to compare tibial and femoral component migration of MC versus CR Persona Knee polyethylene inserts at 12 months using RSA. Secondary aims were to evaluate component migration and patient‐reported outcomes until 24 months follow‐up. We hypothesised that there would be no difference in component migration between the MC and CR inserts.

## PATIENTS AND METHODS

**Figure 1:**
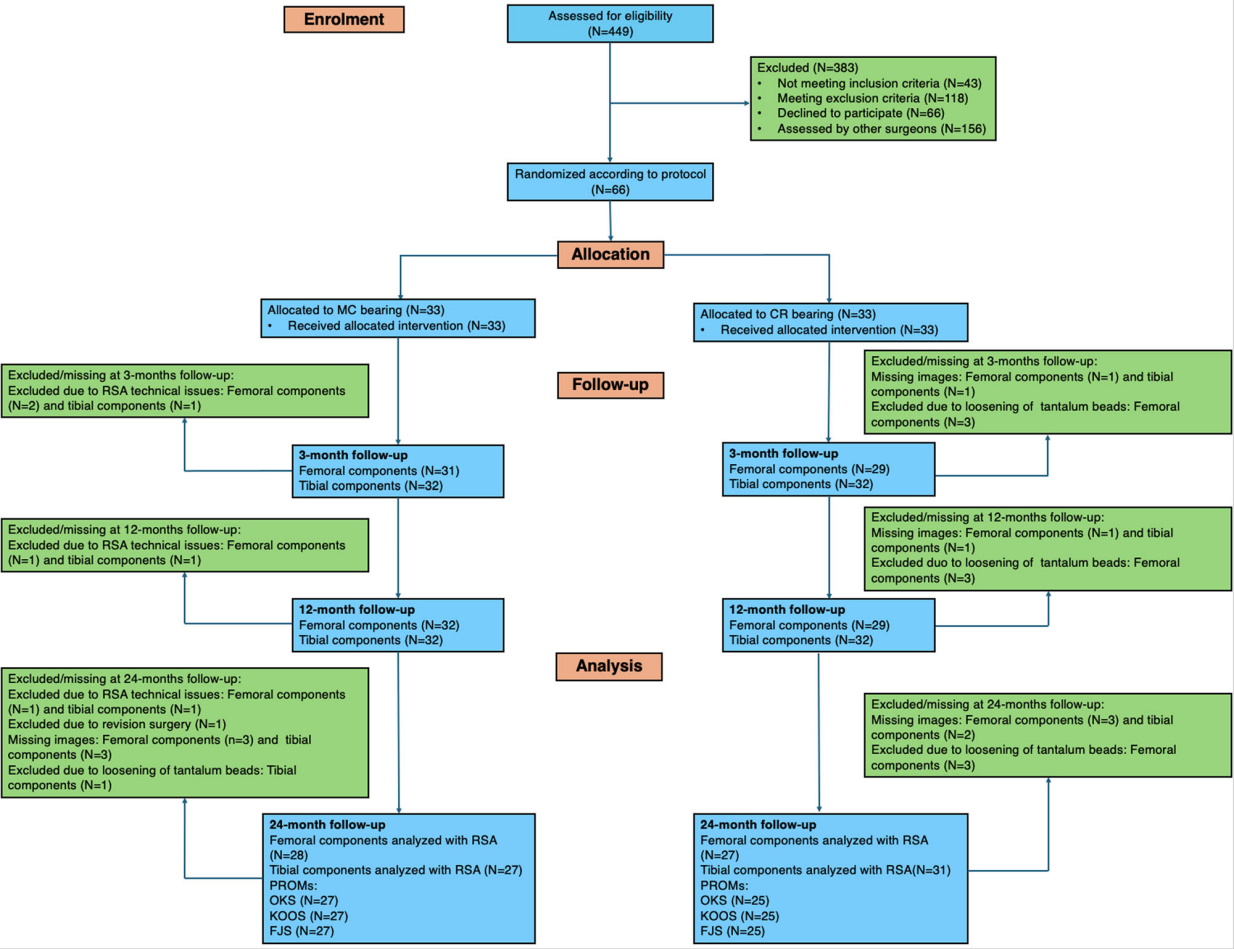
Consort flow diagram.

This double‐blinded prospective randomised study was originally designed to measure knee joint kinematics, with implant migration as a predefined secondary outcome measure [31]. Between 2017 and 2019, 66 patients with primary knee osteoarthritis were enrolled at Regional Hospital Holstebro, Denmark (Figure 1). Inclusion criteria were women and men aged 18–80 years with an indication for cruciate retaining TKA due to osteoarthritis. Exclusion criteria were thigh circumference >60 cm (MRI scanner limitation), implanted metal or pacemakers (MRI contraindication), severely impaired gait, severe fractures or severe malalignment at the knee level, and patients requiring implant augmentation or stem extensions, as these were not candidates for standard TKA.

**Figure 2:**
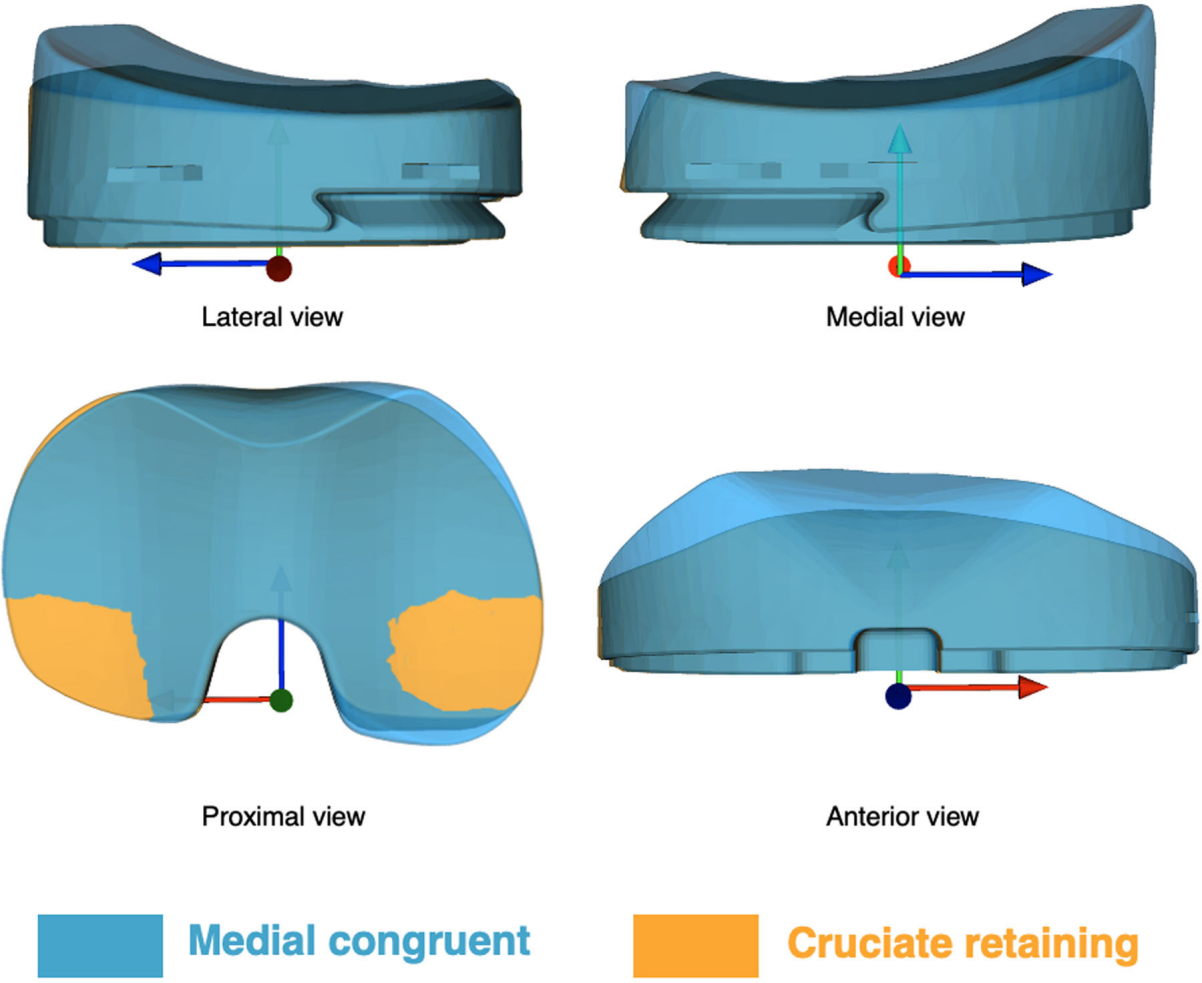
Computer aided design (CAD) illustrations of the differences between the medial congruent (MC) and the cruciate retaining (CR) polyethylene insert.

Patients were randomised in blocks of 10 using concealed opaque envelopes to receive a cruciate retaining TKA (Persona® The Personalized Knee®, Zimmer Biomet, Warsaw, Indiana, USA) with either an MC (Persona® Medial Congruent) or a CR (Persona® Cruciate Retaining) polyethylene insert (Figure 2). All femoral, tibial, and patellar components were compatible with both insert types, making the inserts the only variable between groups. The patients were blinded to group allocation, and data analysis was performed prior to unblinding.

### Surgical procedure

**Figure 3:**
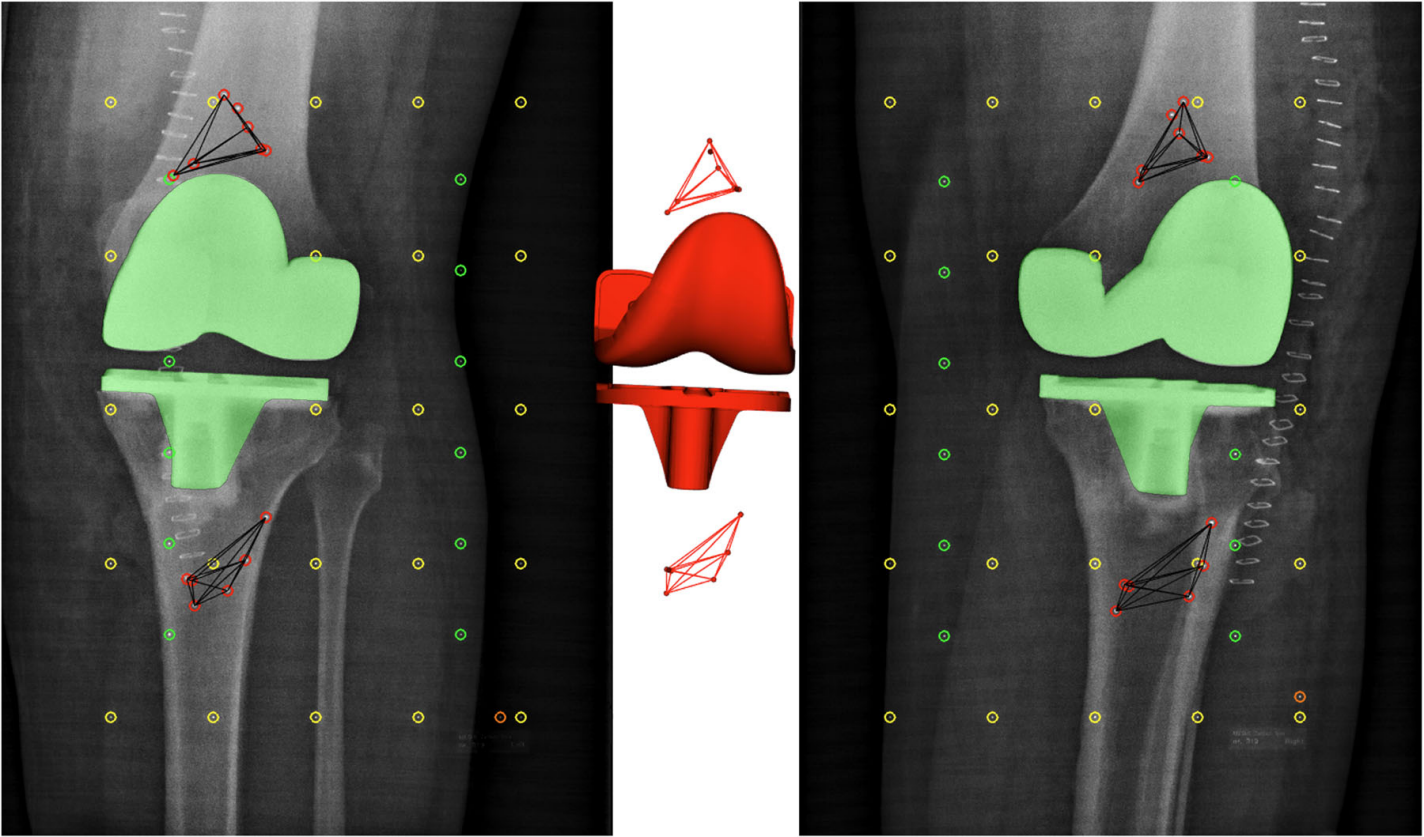
Radiosterometry (RSA) analysis showing calibration box markers (fiducials = yellow, controls = green) and centrally the three‐dimensional (3D) computer aided design (CAD) surface model (red) with its silhouette projection (green) on the RSA image. The tantalum beads are marked on the RSA images (red) with connected lines (black). Their 3D representations (red) are illustrated centrally.

All procedures were performed by three experienced knee arthroplasty surgeons using a standardised technique with an anterior midline incision and a medial parapatellar arthrotomy. Surgery followed the manufacturer's guidelines with a proximal tibial resection aligned with the patient's native tibial slope, using mechanical alignment principles and a 7‐degree cutting guide [2]. All patients received a CR femoral implant and a tibial component paired with either an MC or CR polyethylene insert. Patella resurfacing with all polyethylene patella implants was used in all patients. All components were cemented. Due to a departmental product transition during the study period, unintendedly three different cement types were used. Refobacin‐Optipac® bone cement (Zimmer Biomet, Warsaw, Indiana, USA) in 44 patients, Palacos‐closed bone cement (PALACOS® R + G, Heraeus Medical GmbH, Weirheim, Germany) in nine, and Palacos‐open (PALACOS® MV, Heraeus Medical GmbH, Weirheim, Germany) in thirteen. A minimum of six tantalum bone markers were inserted in both the femoral and tibial bone during surgery (Figure 3). Postoperative rehabilitation and discharge followed standard clinical protocols.

### Radiostereometric analysis (RSA)

The study was conducted per the RSA guidelines [17]. Baseline RSA images were obtained supine on the first postoperative day (after allowance of full weight bearing) and the operated knee secured in a foam positioner. Follow‐up RSA images were captured at 3‐, 12‐, and 24 months. The RSA setup utilised a direct digital synchronised radiostereometric system (AdoraRSA suite, NRT X‐RAY A/S, Hasselager, Denmark) equipped with digital static CXDI‐70C detectors (Canon, Tokyo, Japan). The X‐ray tubes were positioned at a 40‐degree angle, and a uniplanar carbon calibration box (Box 19 Medis Specials, Leiden, The Netherlands) was used for all recordings.

Model‐based RSA version 4.2015 software (RSAcore, Leiden, The Netherlands) was used for image calibration, marker registration, and the final migration analysis. The computer‐aided design (CAD) models of the implant metal components (Zimmer Biomet, Warsaw, Indiana, USA) were analysed utilising AutoRSA software (AutoRSA Research Group, Aarhus University Hospital, Denmark) by minimising the normalised correlation between the Sobel gradient filtered images of the silhouette projection of the CAD model and the stereoradiographs [6, 32]. Evaluation of implant migration until 24 months follow‐up used the postoperative RSA as the reference, and analysis was performed using the coordinate system of the tibial implant. Translations were classified: x‐translation (+medial/−lateral), y‐translation for tibia (+liftoff/−subsidence), y‐translation for femur (+subsidence/−liftoff), z‐translation (+anterior/−posterior), and total translation TT using Pythagorean theorem following the established guidelines [17, 44]. Rotational movements were expressed as x‐rotation (+anterior tilt/−posterior tilt), y‐rotation (+internal/−external), z‐rotation for tibia (+varus/−valgus) and z‐rotation for femur (+valgus/−varus), with total rotation (TR) determined using Pythagorean theorem. Maximal total point motion (MTPM) was the translation vector for the point within the CAD model demonstrating the greatest motion. Stable fixation was defined as migration (MTPM) < 0.2 mm between 12 and 24 months [33, 37].

Marker distribution stability was assessed by the mean error (ME) of rigid body fitting with an upper limit of 0.35 mm, and the condition number (CN) with an upper limit of 150. For the tibial component, the mean ME was 0.16 (SD 0.08; range 0.02–0.35), and the mean CN was 51.5 (SD 40.2; range 17–275). One patient had a CN > 150, the marker model was non‐linear, had a ME of 0.10, and had five markers matched in all scenes. We decided to include the data. For the femoral component, the mean ME was 0.18 (SD 0.08; range 0.02–0.34), and the mean CN was 71.1 (SD 34.2; range 30–205). Two patients had a CN > 150; the specific marker models were non‐linear, had an ME of 0.17 and 0.12, respectively, and both had three markers. We decided to include the data. Double examinations were conducted at the 12‐month follow‐up (*N* = 55).

### Patient‐reported outcome measures and complications

Patient‐reported outcome measures (PROMs) were evaluated preoperatively and at the 12‐ and 24‐month follow‐up using the Oxford Knee Score (OKS) (range 0‐48, 48 best, minimal important change (MIC) 8) [13], Forgotten Joint Score (FJS) (range 0–100, 100 best, MIC 14) [13], and Knee Osteoarthritis Outcome Score (KOOS) (range 0–100, 100 best, MIC range 6–18) [14, 22, 25, 28, 35, 36]. The 12‐month follow‐up results have already been published by Petersen et al. [31]. Additionally, any postoperative complications and revisions occurring within the 24‐month follow‐up period were documented.

### Sample size

The original sample size was determined to detect differences in knee kinematics at the 12‐month follow‐up measured with dynamic RSA, with a sample size of 29 patients in each group [31]. In this secondary study, we determined that with 29 patients in each group at the 12‐month follow‐up, we could potentially detect an effect difference of 0.3 mm in MTPM, with a power of 80%, a significance level of 5%, and an SD of 0.4 [27].

### Data assessment and statistical analysis

The primary outcome was tibial and femoral component migration (MTPM and signed migrations) at the 12‐month follow‐up. Secondary outcomes were component migration and correlations between polyethylene insert type and PROMs until 24 months follow‐up. A paired t‐test was used to compare PROMs at baseline with those at the 24‐month follow‐up. We conducted a subanalysis on the influence of the cement types on migration. A repeated measures mixed model was employed to analyse the primary and secondary outcomes, with the implant group serving as independent factors. The QQ plot of residuals and the fitted versus residuals plots confirmed that the assumption of linearity was met, and no outliers were found. Comparison of proportions between groups was conducted using Pearson's chi‐squared test. The mixed model accommodates missing data by excluding patients only when all observations for that patient are missing. We assumed the missing data points to be missing at random. Thus, all patients could be included in the statistical analysis, ensuring the best statistical power. Statistical significance was defined as *p* < 0.05 and a power of 80%. All statistical analyses were conducted using Stata IC version 18.0.

## RESULTS

### Demographics and follow‐up

Thirty‐three patients were included in each group. Baseline characteristics are presented in Table 1. In total, seven patients could not be analysed (MC, *N* = 5 and CR, *N* = 2) with RSA at 24 months due to technical issues or missing images (Figure 1).

**Table 1:** Demographics.

	MC	CR
Sex (*n*) (female/male)	13/20	13/20
Side (*n*) (left/right)	21/12	15/18
Age (years) (mean, 95% CI)	64.8 (61.8–67.9)	62.0 (59.2–65.9)
Body mass index (kg/m^2^) (mean, 95% CI)	29.0 (27.2–30.8)	29.2 (27.6–30.7)

Abbreviations: CI, confidence interval; CR, cruciate retaining; MC, medial congruent.

### Radiostereometric analysis

#### MTPM tibial component

**Figure 4:**
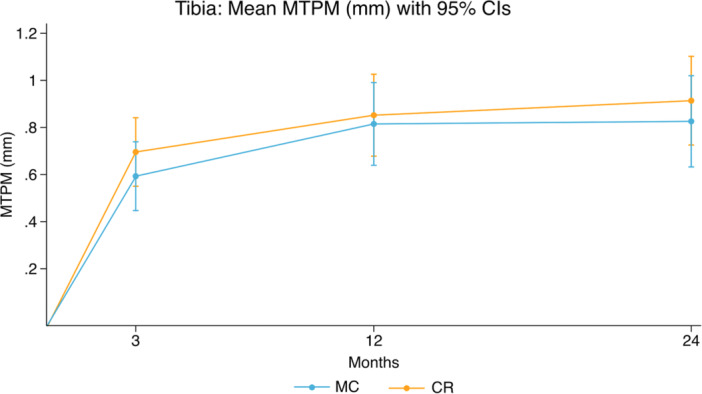
Tibial component mean MTPM (mm) with 95% confidence intervals. CR, cruciate retaining; MC, medial congruent; MTPM, maximal total point motion.

At 12‐month follow‐up, the mean difference in tibial component MTPM between groups was 0.04 mm (95% CI: −0.21 to 0.28) (Figure 4 and Table 2). Adjusted for cement type, the mean group difference at 12 months was 0.04 mm (95% CI: −0.20 to 0.29).

**Table 2:** MTPM and signed migrations for MC and CR tibial components.

	MC	CR		
MTPM (mm)	Mean + 95% CI^a^	Mean + 95% CI^a^	Mean diff + 95% CI^a^	*p* value*
3 months	0.59 (0.45–0.74)	0.70 (0.55–0.84)	−0.10 (−0.31 to 0.10)	0.329
12 months	0.81 (0.64–0.99)	0.85 (0.68–1.03)	−0.04 (−0.28 to 0.21)	0.769
24 months	0.83 (0.63–1.02)	0.91 (0.73–1.10)	−0.09 (−0.36 to 0.18)	0.524
**x‐Translation (mm) medial (+)/lateral (−)**				
3 months	0.01 (−0.07 to 0.09)	−0.05 (−0.13 to 0.03)	0.06 (−0.06 to 0.17)	0.347
12 months	−0.06 (−0.17 to 0.06)	−0.03 (−0.15 to 0.08)	−0.03 (−0.19 to 0.13)	0.743
24 months	−0.08 (−0.21 to 0.06)	−0.03 (−0.16 to 0.10)	−0.05 (−0.24 to 0.14)	0.627
**y‐Translation (mm) liftoff (+)/subsidence (−)**				
3 months	0.01 (−0.04 to 0.05)	0.06 (0.01–0.10)	−0.05 (−0.12 to 0.01)	0.114
12 months	0.03 (−0.03 to 0.09)	0.08 (0.02–0.14)	−0.04 (−0.13 to 0.04)	0.291
24 months	0.03 (−0.03 to 0.09)	0.10 (0.03–0.16)	−0.07 (−0.15 to 0.02)	0.151
**z‐Translation (mm) anterior (+)/posterior (−)**				
3 months	0.00 (−0.12 to 0.12)	0.05 (−0.06 to 0.17)	−0.05 (−0.22 to 0.12)	0.544
12 months	−0.07 (−0.22 to 0.08)	−0.07 (−0.22 to 0.08)	−0.01 (−0.22 to 0.21)	0.955
24 months	−0.10 (−0.25 to 0.05)	−0.09 (−0.23 to 0.06)	−0.02 (−0.23 to 0.19)	0.867
**x‐Rotation (°) anterior tilt (+)/posterior tilt (−)**				
3 months	0.00 (0.17–0.16)	0.08 (−0.08 to 0.25)	−0.09 (−0.32 to 0.15)	0.464
12 months	−0.07 (−0.28 to 0.14)	−0.01 (−0.22 to 0.20)	−0.06 (−0.35 to 0.24)	0.699
24 months	−0.14 (−0.37 to 0.09)	0.02 (−0.21 to 0.24)	−0.16 (−0.47 to 0.16)	0.338
**y‐Rotation (°) internal rotation (+)/external rotation (−)**				
3 months	0.08 (−0.08 to 0.25)	−0.11 (−0.27 to 0.05)	0.19 (−0.04 to 0.42)	0.098
12 months	−0.10 (−0.31 to 0.10)	−0.18 (−0.39 to 0.02)	0.08 (−0.21 to 0.37)	0.587
24 months	−0.11 (−0.33 to 0.10)	−0.07 (−0.28 to 0.14)	−0.04 (−0.34 to 0.26)	0.777
**z‐Rotation (°) varus tilt (+)/valgus tilt (−)**				
3 months	0.01 (−0.09 to 0.11)	0.05 (−0.05 to 0.14)	−0.04 (−0.17 to 0.10)	0.612
12 months	0.06 (−0.07 to 0.18)	0.03 (−0.09 to 0.15)	0.03 (−0.15 to 0.20)	0.767
24 months	0.08 (−0.07 to 0.23)	0.04 (−0.11 to 0.18)	0.04 (−0.16 to 0.25)	0.686

Abbreviations: CI, confidence interval; CR, cruciate retaining; MC, medial congruent; MTPM, maximal total point motion.

^a^ Estimated marginal means by mixed model analysis.

* Refers to mean difference, significance level < 0.05.

#### Signed migrations tibial component

No statistically significant differences in tibial component signed translations or rotations were observed between the groups at any time point (Table 2).

#### MTPM femoral component

**Figure 5:**
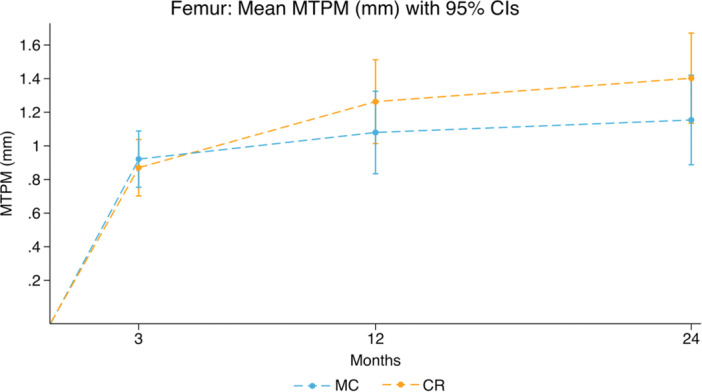
Femoral component mean MTPM (mm) with 95% confidence intervals. CR, cruciate retaining; MC, medial congruent; MTPM, maximal total point motion.

At the 12‐month follow‐up, the mean difference in femoral component MTPM was 0.18 mm (95% CI: −0.17 to 0.53) (Figure 5 and Table 3).

**Table 3:** MTPM and signed migrations for MC and CR femoral components.

	MC	CR		
MTPM (mm)	Mean + 95% CI^a^	Mean + 95% CI^a^	Mean diff + 95% CI^a^	*p* value*
3 months	0.92 (0.75–1.09)	0.87 (0.70–1.04)	0.05 (−0.19 to 0.29)	0.674
12 months	1.08 (0.83–1.32)	1.26 (1.01–1.51)	−0.18 (−0.53 to 0.17)	0.304
24 months	1.15 (0.89–1.42)	1.40 (1.13–1.67)	−0.25 (−0.63 to 0.13)	0.197
**x‐Translation (mm) medial (+)/lateral (−)**				
3 months	−0.13 (−0.24 to −0.03)	0.05 (−0.05 to 0.15)	−0.18 (−0.33 to −0.04)	0.013
12 months	−0.13 (−0.26 to 0.00)	0.07 (−0.06 to 0.20)	−0.20 (−0.39 to −0.02)	0.034
24 months	−0.17 (−0.22 to −0.02)	−0.01 (−0.17 to 0.15)	−0.16 (−0.38 to 0.06)	0.156
**y‐Translation (mm) subsidence (+)/liftoff (−)**				
3 months	0.10 (0.06–0.14)	0.09 (0.05–0.13)	0.01 (−0.05 to 0.07)	0.752
12 months	0.14 (0.09–0.19)	0.13 (0.07–0.18)	0.01 (−0.06 to 0.09)	0.745
24 months	0.20 (0.13–0.27)	0.17 (0.10–0.24)	0.03 (−0.07 to 0.13)	0.525
**z‐ Translation (mm) anterior (+)/posterior (−)**				
3 months	0.02 (−0.16 to 0.19)	−0.04 (−0.22 to 0.14)	0.06 (−0.19 to 0.30)	0.660
12 months	−0.10 (−0.36 to 0.16)	−0.15 (−0.42 to 0.12)	0.05 (−0.33 to 0.42)	0.800
24 months	−0.32 (−0.59 to −0.04)	−0.22 (−0.50 to 0.06)	−0.10 (−0.49 to 0.29)	0.631
**x‐Rotation (°) anterior tilt (+)/posterior tilt (−)**				
3 months	0.04 (−0.14 to 0.23)	0.18 (−0.01 to 0.37)	−0.14 (−0.40 to 0.13)	0.309
12 months	0.17 (−0.11 to 0.46)	0.17 (−0.12 to 0.46)	0.00 (−0.41 to 0.40)	0.994
24 months	0.40 (0.11 to 0.69)	0.32 (0.03 to 0.61)	0.08 (−0.33 to 0.49)	0.706
**y‐Rotation (°) internal rotation (+)/external rotation (−)**				
3 months	0.32 (0.13–0.51)	0.08 (−0.12 to 0.27)	0.24 (−0.03 to 0.51)	0.081
12 months	0.20 (−0.07 to 0.48)	−0.06 (−0.34 to 0.21)	0.27 (−0.12 to 0.65)	0.178
24 months	0.30 (0.05–0.56)	0.09 (−0.16 to 0.35)	0.21 (−0.15 to 0.57)	0.255
**z‐Rotation (°) valgus tilt (+)/varus tilt (−)**				
3 months	−0.06 (−0.17 to 0.05)	0.02 (−0.08 to 0.13)	−0.08 (−0.24 to 0.07)	0.289
12 months	−0.12 (−0.24 to 0.00)	0.01 (−0.11 to 0.14)	−0.13 (−0.31 to 0.04)	0.139
24 months	−0.09 (−0.23 to 0.06)	−0.01 (−0.16 to 0.14)	−0.08 (−0.28 to 0.13)	0.471

Abbreviations: CI, confidence interval; CR, cruciate retaining; MC, medial congruent; MTPM, maximal total point motion.

^a^ Estimated marginal means by mixed model analysis.

* Refers to mean difference, significance level < 0.05.

#### Signed migrations femoral component

A statistically significant difference was found only in x‐translation for the femoral component at 3 months and 12 months (Table 3).

#### Stable fixation

Continuous migration (MTPM > 0.2 mm) was exhibited for 16 tibial components (7/28 MC and 9/30 CR (*p* = 0.67)) and 22 femoral components (12/28 MC and 10/28 CR (*p* = 0.58)). No differences in continuous migration proportions were found between the three cement groups for either the tibial (*p* = 0.16) or femoral (*p* = 0.33) components.

#### Sub‐analysis of cement types

The distribution of cement types was the same between groups. Overall, 44 patients (MC: *N* = 23, CR: *N* = 21) received the Refobacin‐Optipac bone cement, nine patients (MC: *N* = 4, CR: *N* = 5) received the Palacos‐closed bone cement, and 13 patients (MC: *N* = 6, CR: *N* = 7) received the Palacos‐open bone cement. Due to the use of three different bone cement types, we conducted a subanalysis on the entire cohort to assess if the type of cement could affect the comparison of implant migration between the intervention groups. At 24 months, the tibial components inserted with Refobacin‐Optipac bone cement had a higher MTPM of 0.99 mm (95%CI: 0.84–1.15) compared to MTPM of 0.53 mm (95%CI: 0.17–0.89) for the tibial components inserted with Palacos‐closed bone cement. The mean difference was 0.47 mm (95%CI: 0.07–0.86, *p* = 0.02). Thus, adjustment for cement type was included in the primary analysis of the tibial component. At all other time points for the tibial component, there was no difference in MTPM between cement types (*p* > 0.07). There was no difference in MTPM at any time point for the femoral component between cement types (*p* > 0.11). Thus, no adjustment for cement type was included in the primary analysis of the femoral MC versus CR component migration.

#### Double examinations

Precision based on double examination was evaluated as acceptable/good compared to other studies on TKA implant migration (Table 4).

**Table 4:** RSA double‐examination measurement error for the tibial component (*n* = 55).

	Translations (mm)				Rotations (°)				
Axis	*x*	*y*	*z*	TT^a^	*x*	*y*	*z*	TR^b^	MTPM (mm)
Mean dif.	0.01	0.00	−0.02	0.01	−0.01	0.04	0.01	0.06	0.06
SD dif.	0.06	0.04	0.15	0.11	0.21	0.45	0.06	0.26	0.23
CR (±1.96 * SD dif.)	0.11	0.09	0.29	0.22	0.40	0.88	0.13	0.51	0.44

RSA double‐examination measurement error for the femoral component (*n* = 53)									
	Translations, mm				Rotations°				MTPM (mm)
Axis	*x*	*y*	*z*	TT^a^	*x*	*y*	*z*	TR^b^	
Mean dif.	0.04	−0.01	0.02	−0.07	0.03	−0.04	0.01	−0.12	−0.11
SD dif.	0.19	0.06	0.35	0.27	0.54	0.49	0.10	0.50	0.49
CR (±1.96 * SD dif.)	0.37	0.12	0.69	0.53	1.06	0.96	0.19	0.99	0.96

*Note*: Mean dif. represents the systematic measurement error. SD dif. represents the random variation (precision). Coefficient of repeatability (CR) (±1.96 * SD dif.) represents the expected clinical precision.

Abbreviations: MTPM, maximal total point motion; RSA, radiosterometry; SD, standard deviation.

^a^ Total translation (TT) was calculated using the Pythagorean theorem.

^b^ Total rotation (TR) was calculated using the Pythagorean theorem.

### Patient‐reported outcome measures and complications

There were no statistically significant differences between the PROMS for the two groups at baseline or the 24‐month follow‐up. Both groups improved above the MIC for both OKS, all KOOS‐subscores and for FJS [13, 25]. Mean combined improvements from baseline to the 24‐month follow‐up are presented in Table 5.

**Table 5:** PROM scores at baseline and at 24 months combined for the entire cohort (*N* = 52).

PROM	Baseline, Mean + 95% CI	24 months, Mean + 95% CI	Mean difference + 95% CI^a^
OKS	23.5 (21.8–25.2)	37.9 (35.8–40.0)	14.4 (12.4–16.5)
KOOS Pain	44.1 (39.1–48.5)	80.3 (75.8–84.8)	36.2 (30.9–41.5)
KOOS ADL	52.6 (48.5–56.6)	81.3 (74.1–85.5)	28.7 (24.2–33.2)
KOOS Sport	16.1 (11.9–20.2)	44.9 (37.3–52.5)	28.8 (22.5–35.2)
KOOS Symptoms	48.8 (43.9–53.7)	73.6 (67.8–79.4)	24.9 (19.0–30.7)
KOOS QOL	27.5 (24.1–30.9)	63.5 (57.1–69.8)	35.9 (29.7–42.2)
FJS	16.8 (12.4–21.2)	54.5 (46.2–62.7)	37.7 (29.8–45.6)

*Note*: *N* = 52 including patients who completed PROMs at both baseline and 24 months follow‐up.

Abbreviations: FJS, Forgotten Joint Score; KOOS ADL, Knee Injury And Osteoarthritis Outcome Score Activities of Daily Living; KOOS QOL, KOOS Quality of Life; PROMs, patient reported outcome measurements.

^a^ Paired t‐test.

The mean difference in OKS between groups at baseline was −1.70 (95% CI: −4.71 to 1.31, *p* = 0.27), and at 24 months, it was −1.62 (95% CI: −5.17 to 1.93, *p* = 0.37). For the five KOOS sub‐scores, there was no difference at baseline (*p* > 0.27), or at the 24‐month follow‐up (*p* > 0.54) between groups with mean differences at the 24‐month follow‐up of −0.87 (95% CI: −5.17 to 1.93), 1.30 (95% CI: −6.12 to 8.72), 2.80 (95% CI: −10.5 to 16.1), 3,25 (95% CI: −7.16 to 13.67) and −0,81 (95% CI: −12.38 to 10.76), for KOOS Pain, KOOS Activities of Daily Living (ADL), KOOS Sport, KOOS Symptoms and KOOS Quality of Life (QOL) respectively. The mean difference in FJS at baseline and 24 months was −0.25 (95% CI: −7.55 to 7.05, *p* = 0.95) and − 0.22 (95% CI: −15.20 to 14.79, *p* = 0.98), respectively.

### Complications and revisions

One patient from the MC group had a TKA revision surgery to a knee with tibia stem lengthening at 24 months follow‐up due to pain and restricted ROM in both flexion and extension. There were no infections, component loosening, or peri‐prosthetic fractures.

## DISCUSSION

The main finding of the present study was that the Persona TKA system with an MC or a CR polyethylene insert had similar tibial and femoral component MTPM migration. There were indications of more lateral migration for femoral components in the MC group, but the migrations were small and likely not clinically relevant. Thus, a more constrained MC polyethylene insert did not seem to affect implant migration up until 24 months and, as such, supports the safety of using MC polyethylene inserts.

### MC polyethylene insert—Influence on migration

The main concern of the constraint of an MC insert is that it induces higher strain and stress to the implant‐bone interface during gait and other exercises [1, 31]. However, anatomical knee designs are also designed to provide native knee kinematics, which may further improve knee stability [4, 42]. Christensson et al. also investigated component migration of mechanically aligned cemented Persona Knee (Zimmer Biomet) in 60 patients, and at 12 months follow‐up they found 0.61 mm and 0.59 mm MTPM for the tibial components and 0.58 mm and 0.73 mm MTPM for the femoral components in MC and CR polyethylene inserts, respectively [7]. Mortensen et al. investigated tibial component migration in 60 patients with a mechanically aligned and cemented Persona Knee (Zimmer Biomet) and found a mean 12 month MTPM of 0.71 mm and 0.71 mm for the MC and CR polyethylene inserts, respectively [26]. Nivbrant et al. showed stable tibial fixation in 35 patients for a cemented highly congruent medial pivot TKA SAIPH implant (MatOrtho) with a median MTPM of 0.44 mm at 12 months follow‐up [29]. Koster et al. compared an asymmetric (Persona PS TKA) and a symmetric (NextGen LPS TKA) posterior stabilised knee in 75 patients. Both systems were mechanically aligned and cemented knee systems. They found similar migration of 0.93 mm and 1.00 mm for the tibial components and 1.04 mm and 1.14 mm for the femoral components, of the asymmetric and symmetric polyethylene inserts respectively [18]. Øhrn et al. [47], investigated the migration of the tibial component of the cemented medial pivot GMK Sphere implant in 31 patients and found a mean MTPM of 1.30 mm at 12 months follow‐up. They concluded that it was a little higher than expected. The GMK Sphere implant is more congruent than the MC insert in the present study, which may explain the difference. In line with these studies, the present study found no clinically relevant difference in tibial and femoral component migration comparing an MC and a CR polyethylene insert. Thus, regarding implant fixation, it seems generally safe to use highly congruent implant designs such as the MC insert.

### Acceptable component migration

Studies have indicated a good 5‐year survival of 99% for the Persona Knee [23]. At 12‐month follow‐up, we found a mean tibial component MTPM of 0.81 mm for the MC group and 0.85 mm for the CR group. This is slightly more than the acceptable range for 12 months MTPM for cemented tibial components of 0.5 mm, but less than the unacceptable migration limit of 1.6 mm according to the migration limits presented in the meta‐analysis of tibial component migration by Pijls et al. [33]. We observed continuous migration for 16/58 tibial components, equally distributed between MC and CR groups. Other studies on cemented TKAs have reported proportions of continuous migration >25%, similar to our study [20, 40].

The literature on femoral component migration is limited, and there are no validated limits for acceptable femoral component migration. However, other studies investigating both tibial and femoral component migration are indicative of similar MTPM magnitudes between tibial and femoral components [7, 18, 43]. At 12 months, we observed a mean femoral component MTPM of 1.08 mm for the MC group and 1.26 mm for the CR group. Christensson et al. [7] studied the same type of implant and found a mean MTPM at 12 months of 0.58 mm for the MC group and 0.73 mm for the CR group.

### Bone cement

Several studies have assessed the effect of different types of bone cement on the fixation of knee arthroplasty and hip arthroplasty components [3, 8, 16, 19, 45]. These studies indicated no or minimal differences between different cement types [8, 16, 45]. In our study, nine patients of whom seven were analysed at the 24‐month follow‐up, received the Palacos R + G bone cement. This group exhibited the lowest tibial component migration with a mean MTPM of 0.53 mm at 24 months. Christensson et al. used the Palacos R + G bone cement in all their patients and found a 12‐month mean tibial component MTPM of 0.61 mm and 0.59 mm for the MC and CR groups, respectively. This suggests that the difference in cement types between the current study and the Christensson study might explain the overall lower component migration seen in their study. Mortensen et al. used Refobacin Optipac® bone cement in all patients and reported 12 months of tibial component migration, similar to the present study for both MC and CR.

### Timing of baseline RSA imaging

Most component migration happens within the first month or even the first few weeks for both cemented and cementless components. Potentially, there is a loading‐induced migration of the implant with the first weight bearing and mobilisation after surgery [30]. Therefore, when investigating and comparing implant migration between studies, it is important to consider the timing of the reference RSA image [17, 44]. Teeter et al. captured reference RSA images within the first two weeks after surgery, and Henricson et al. captured the reference RSA image at a mean of 4 days postoperatively and after weight bearing, whereas we obtained ours on the first postoperative day. Therefore, this timing difference may partially explain the higher component MTPM in our study.

### Patient‐reported outcome measures

High patient satisfaction of 95% at 12 months has been reported for the Persona Knee [23]. We found clinically relevant improvements from preoperative until 24 months follow‐up. This aligns with other studies investigating clinical outcomes for the Persona Total Knee System [7, 10, 23, 24]. Furthermore, we did not find a difference in OKS, FJS and KOOS scores between the MC and CR groups. One study indicated better PROM outcomes on the FJS‐12 score, a higher rate of satisfaction, and a lower rate of patients who were dissatisfied with the MC insert than with the CR insert [9]. Other studies comparing the MC and CR components indicated no difference in PROMs between the two groups [7, 12, 26, 31].

### Strengths and limitations

A major strength of this study is the randomised double‐blinded design, which ensures the best conditions for minimising confounding and bias. Furthermore, RSA is a very precise method for measuring implant migration to compare new implant designs against gold‐standard designs [15, 38, 46]. The use of three different types of bone cement during the study period was unintended and is a limitation of the study. However, the distribution of patients with MC and CR polyethylene inserts was similar across the cement groups, mitigating potential bias.

The sample size being determined for dynamic RSA analysis (knee kinematics) as the primary outcome is another limitation [31]. We determined that the sample size was sufficient to find an effect difference of 0.3 mm in MTPM between groups in a static RSA setup. This effect difference was evaluated as clinically relevant. The sample size was insufficient to determine any differences in PROMs between the MC and the CR insert, which is a limitation. The statistically significant differences found in the medial‐lateral translation of the femoral components have minimal magnitude and can likely be attributed to a type 1 error due to multiple testing. The double examinations overall indicate the precision of our study to be good compared to other studies on TKA implant migration [33].

## CONCLUSION

Overall, the results suggest no clinically meaningful differences in migration between the MC and CR groups suggesting that increased constraint of the MC polyethylene insert did not affect component fixation. The present results indicate that a more constrained MC polyethylene insert did not seem to affect implant migration up until 24 months and, as such, support the safety of using MC polyethylene inserts.

## AUTHOR CONTRIBUTIONS

All authors contributed to the study conceptualisation, design, material preparation, and data collection. Image and data analysis were performed by Carl Christian Holkgaard Burvil, Emil Toft Petersen, and Karina Nørgaard Linde. The first draft of the manuscript was written by Carl Christian Holkgaard Burvil, and all authors commented and helped revise the manuscript. All authors read and approved the final manuscript.

## CONFLICT OF INTEREST STATEMENT

The authors declare no conflicts of interest.

## ETHICS STATEMENT

The study was approved by the Committee on Biomedical Research Ethics of the Central Denmark Region (1‐10‐72‐303‐16, issued 28 February 2017) and registered with the Danish Data Protection Agency (1‐16‐02‐582‐16, issued 31 October 2016). All patients gave written informed consent. The study was registered with clinical trials ID NCT03633201 Date 2018‐01‐18.

## Data Availability

Requests for data access may be granted on a case‐by‐case basis.
